# Unravelling the relationship between perceived values-congruence with organizational change readiness: A moderated mediation model

**DOI:** 10.3389/fpsyg.2023.1086326

**Published:** 2023-02-22

**Authors:** Jinzhao Deng, Zhihui Cheng, Siqi Qi, Rich Deng

**Affiliations:** ^1^Research Centre of Hubei Enterprise Culture, Hubei University of Economics, Wuhan, China; ^2^School of Accountancy, Hubei University of Economics, Wuhan, China; ^3^Telfer School of Management, University of Ottawa, Ottawa, ON, Canada

**Keywords:** perceived values-congruence, perceived insider status, quality of change communication, organizational change readiness, person-environment interaction

## Abstract

Recent studies have demonstrated that organizations often fail to execute organizational changes effectively due to a lack of their employees’ organizational change readiness (OCR). However, the absence of employees’ OCR is rooted in whether their values align with their organizations. The research aims to clarify when and why employees’ perceived values-congruence with their organizations, supervisors, and workgroups (PVC-O, PVC-S, and PVC-G) helps stimulate their organizational change readiness (OCR). Specifically, it Integrates the self-categorization theory and social information processing theory and proposes a moderated mediation model to investigate the roles of perceived insider status (PIS) and the quality of change communication (QCC). This study gathered a valid sample of 252 employees from six Chinese companies at three different time points, and performed the structural equation modeling and multiple regression to test the proposed research model. The results demonstrate that PVC-O, PVC-S, and PVC-G are all positively related to employees’ PIS, which further promotes their readiness for organizational change. Additionally, QCC strengthens not only the positive effect of employees’ PVC-O and PVC-G (except for PVC-S) on PIS but also the indirect effects of PIS. This study offers valuable implications for practitioners implementing their organizational change practices in China. Moreover, this study can contribute to the organizational change literature by uncovering the underlying mechanism between perceived values-congruence and employees’ OCR in the light of the person-environment interaction.

## 1. Introduction

In recent years, the volatility, uncertainty, complexity, and ambiguity of the current business environment have been on the rise (“VUCA,” see Bennett and Lemoine, 2014), especially after the ongoing COVID-19 pandemic outbreak, which has heightened the urgency of organizational change (Shah et al., 2017; Gfrerer et al., 2021; Roemer et al., 2021). Yet, organizations might not successfully implement change without employees’ readiness for organizational change (Stouten et al., 2018; Hameed et al., 2019; Rafferty and Minbashian, 2019; Rahn et al., 2020). Organizational change readiness (hereinafter “OCR”) refers to “the degree to which individuals are cognitively and emotionally inclined to accept, embrace, and adopt specific change plans to purposefully alter an existing state” (Stevens, 2013). Prior research has identified OCR as a critical component for more effective change implementation (Rafferty et al., 2013; Rahn et al., 2020). Hence, it is of great significance to investigate the antecedents and underlying mechanisms of the formation of employees’ OCR.

Previous research has identified the antecedent variables of organizational change readiness from two main perspectives. From an individual dispositional perspective, scholars have found that personal traits (i.e., self-efficacy, locus of control, coping styles, and openness) affect employees’ OCR (Judge et al., 1999; Augustsson et al., 2017; Naumtseva and Stroh, 2020); whereas from the perspective of change strategies, studies have found that leader or organizational support (Soumyaja et al., 2011; Arneguy et al., 2020), trust and change participation (Shah and Ghulam Sarwar Shah, 2010), and organizational justice (Arneguy et al., 2020) positively impact organizational change readiness.

However, we surprisingly found that extant literature largely overlooked the impact of personal values (i.e., values-congruence) on employees’ OCR, let alone the underlying mechanisms. Despite that, some scholars have highlighted the critical role of values in implementing organizational change (Branson, 2008; Alas, 2009; Rahn et al., 2020) because values serve as the guiding principles for an individual’s attitudes and behaviors. For example, values-conflict might raise employee’s resistance to change, whereas values-congruence might be conducive to change readiness (Bouckenooghe et al., 2014). Moreover, little knowledge exists regarding the boundary conditions (i.e., change communication) on when employees’ perceived values-congruence affects their OCR since change communication is a crucial change strategy that provides employees’ cues for implementing organizational change. Therefore, the present study aims to integrate self-categrication theory and social information theory to investigate when and why perceived values-congruence affects employees’ OCR.

The present study seeks to make three aspects of theoretical contributions. Firstly, we enrich the psychological outcomes research on values-congruence by investigating its impact on perceived insider status (PIS). Secondly, we contribute to a burgeoning stream of research that probes into the underlying mechanism of how employees’ OCR is formed. Specifically, based on self-categorization theory (Turner et al., 1987), we examine the mediating role of PIS, a construct embedded in Chinese circle culture, that played in the relationship between employees’ perceived values-congruence on organizational change readiness. Prior scholars have raised the idea that values-congruence may be beneficial in cultivating employees’ OCR (Alas, 2009; Rahn et al., 2020). However, the empirical evidence is scarce, and little research uncovers the mechanism. Finally, we enrich the boundary conditions research between organizational change readiness and its antecedents. Specifically, drawing on the social information processing theory (Salancik and Pfeffer, 1978), we demonstrate when (i.e., quality of change communication) values-congruence perception is most influential in cultivating PIS and promoting employees’ OCR.

## 2. Theory and hypotheses

A series of stages and complex mechanisms exist in the process of perceived values-congruence to employees’ OCR, and these stages and mechanisms are critical to our insight and the deconstruction of the impact of employees’ perceived values-congruence on their OCR (Lamm et al., 2010; Hoffman et al., 2011; Rahn et al., 2020). Based on the self-categorization theory (Turner et al., 1987), we propose that the stronger the employees’ perceived insider identity, the more likely they are to meet the organization’s expectations and actively embrace and support changes to reduce the risk of contract violations. In line with this reasoning, we argue that employees must go through a mental process of insider identity perception from evaluating employees’ perceived values-congruence to the formation of the corresponding OCR.

Moreover, drawing on the social information processing theory (Salancik and Pfeffer, 1978), we assumed that the employees’ perception of insider identity could be the influence path for values congruence perceptions (i.e., organization, supervisor, and workgroup) on OCR due to differences in the modes of acquisition of change information, the quality and quantity of information, the necessity for organizational change, and the need for a change plan. Significant differences exist in understanding suitability, organizational change capability, and change benefit orientation (Stevens, 2013). High-quality change communication (e.g., having the opportunity to obtain timely, sufficient, and essential change information) can enhance employees’ PIS and also effectively alleviate employees’ change anxiety (Bernerth, 2004; Branson, 2008; Borges and Quintas, 2020). Therefore, this study attempts to adopt the quality of change communication as the contingent condition for employees’ multiple value congruence perceptions affecting OCR through PIS.

### 2.1. The effect of perceived values-congruence on employees’ perceived insider status

Values congruence is an essential concept derived from person-organization fit and characterizes the degree of compatibility and congruence between values (Edwards and Cable, 2009). Perceived values-congruence refers to one’s perception of the degree of conformity or similarity, compatibility, and similarity with the interacting objects (i.e., organizations, supervisors, and workgroups) in terms of values (Kristof-Brown et al., 2005; Rahn et al., 2020). Previous studies have demonstrated that perceived values-congruence can positively impact job satisfaction, organizational identity, and retention (Aldabbas, 2022), reduce change resistance (Smollan and Sayers, 2009), and positively affect employees’ positive change response, change support behavior, and change acceptance (Smollan and Sayers, 2009; Posner, 2010; Rahn et al., 2020).

Drawing on the self-categorization theory, we argue that perceived values-congruence (i.e., congruence with their organization, supervisor, and workgroup) can result in an increase in employees’ perceived insider status (hereinafter PIS). PIS refers to the degree to which employees believe that the organization accepts them, are “insiders,” and can access organizational space and resources (Stamper and Masterson, 2002). The similarity between individuals needs and environmental supply in terms of basic principles, behavioral norms, and value orientations can alter individual cognition and enhance relationship correlation and identity. For example, some scholars found that when employees experience a high degree of similarity and compatibility with organizational values, they were more likely to form a self-concept consistent with the characteristics of organizational prototypes and derive a sense of belonging to a certain group (Wang and Kim, 2013; Aldabbas, 2022).

In the same vein, if individuals and their supervisors display a high degree of similarity in values, individuals will exhibit strong situational adaptability in cognition (Alavi and Gill, 2017). Finally, as per the self-categorization theory, since individuals are nested in and interacting with their workgroups, they are more likely to have feelings of “us,” and tend to form an identity cognition of belonging to a certain group(Hui et al., 2015; Aldabbas, 2022). Hence, based on the above discussion, we propose the following hypotheses:

*Hypothesis 1:* Employees’ perceived values-congruence with their organization (H1a), their supervisors (H1b), and their workgroups (H1c) has a positive effect on PIS.

### 2.2. The mediating role of perceived insider status

Organizational change readiness denotes employees’ attitudes and behavioral intentions toward organizational change and represents employees’ acceptance of organizational change in attitude, belief, cognition, emotion, and state of readiness for change (Stevens, 2013). Although some scholars posited the critical role in employing the“acquaintance effect” to promote employees’ OCR (Bouckenooghe et al., 2014; Seggewiss et al., 2019), few scholars have directly investigated the relationship between PIS and OCR, with the exception of some related research providing us with valuable insights. For example, Xiao et al. (2020) believe that the partnership and community formed due to the interaction between people and organizations are conducive to forming identity attachments for employees, which is particularly meaningful when the organization is going through a particular period, especially one of upheaval or turmoil. In such a situation, employees’ willingness to keep the organization in sync can be enhanced, and they will participate in its weal and woe. We thus infer that the stronger the PIS, the higher the employee’s level of organizational change readiness.

According to the self-categorization theory (Turner et al., 1987), an individual will form the boundaries of “internal and external groups” after a self-categorization process. Thus, the status of the other individuals in the organization and the overall status of the organization become more closely related to the status of the self, prompting individuals to be more inclined to display behaviors beneficial to the organization, to enhance the performance of the organization, and demonstrate the value of the self as a part of the organizational whole (Tang et al., 2021). Thus, members with strong insider identity perception will be motivated to promote the performance of the organization or group to which the identity belongs, and the impact of employees’ PIS on their OCR is rooted in this logic. The higher the employee’s perceived congruence with the organization or group values, the more meaningful it would be to enhance that employee’s awareness of organizational and group identity, and the stronger her/his PIS; when employees use the organization and group to define themselves, the performance of the organization and group will reflect their self-worth (Hui et al., 2015). In the context of change, planned organizational change represents the official will of the group, which aims to augment the potential performance and promote the improved development of the organization. Given their interlinked relationship with the organization or workgroup, employees with a high sense of insider identity are more likely to become advocates and agents of change.

In the same rationale, it is evident that the incremental fit of supervisor-employee values helps improve the situational adaptability of individual cognition (Santhidran et al., 2013; Rahn et al., 2020) and prompts subordinates to form a positive psychological state as insiders. In the context of change, the team supervisor is not only the “Avatar” of the organization but also the initiator and promoter of change. Individuals with high PIS preoccupy themselves with accurately observing and understanding their intermediate supervisor’s change intentions (Bakari et al., 2017). In other words, Employees must be consistent with their supervisor to actively respond to the supervisor’s call for change. Hence, we hypothesize as follows:

*Hypothesis 2:* Employees’ PIS positively affects OCR.

*Hypothesis 3:* Employees’ PIS mediates the relationship between employees’ perceived values-congruence with their organization (H3a), their supervisors (H3b), and their work groups (H3c) and OCR.

### 2.3. The moderating role of quality of change communication

The quality of change communication is an essential construct in studying organizational change strategies, which refers to the depth, breadth, and importance of organizational change information obtained by employees during the change process (Rogiest et al., 2015). Prior research has highlighted that the success of organizational change depends, at least in part, on the quality of the information the organization provides (Tanner and Otto, 2016; Güntner et al., 2021). Accordingly, we deduce that how the change-related information delivers to the employees affects whether the organizational change readiness forms.

According to the social information processing theory (Salancik and Pfeffer, 1978), individuals, as adaptive organisms, will resort to social clues to adjust their attitudes and behaviors. Hence, we propose that the degree of after-effects on employees’ perceived values-congruence on its outcomes may differ with different qualities of change communication. Specifically, employees obtain more comprehensive and rich change information, which helps them understand the change in its entirety, reduces the pressure of change, and improves their sense of control over the change (Bernerth, 2004), which affords employees a more internal identity construction. Therefore, high-quality change communication strengthens the relationship between employees’ perception of multiple values congruence and PIS, which further influences their OCR. Conversely, when the quality of change communication is low, individuals who find it difficult to obtain basic change information are more likely to feel excluded and would tend to self-identify as outsiders, increasing their psychological detachment (Kwahk and Kim, 2008; Thakur and Srivastava, 2018), and thereby inhibiting the effect of perceived values-congruence on PIS. In line with this reasoning, we hypothesize as follows:

*Hypothesis 4:* The quality of change communication moderates the relationship between perceived values-congruence and PIS. Specifically, employees’ perceived values-congruence with their organization (H4a), their supervisors (H4b), and their workgroups (H4c) have a more substantial positive effect on PIS when the quality of change communication is high rather than when it is low.

### 2.4. Moderated mediation

Hypothesis 4 posits an interplay between perceived values-congruence and the quality of change communication on employees’ PIS. Hypothesis 3 predicts that PIS mediates the association between perceived values-congruence and employees’ OCR. In line with the mediated moderation of Edwards and Lambert (2007), we propose a moderated mediation of perceived values-congruence on employees’ OCR. Thus, we predict that,

*Hypothesis 5:* The quality of change communication can moderate the indirect effects of PIS between employees’ perceived values-congruence with their organization (5a), their supervisors (5b), and their work groups (5c) on OCR. Specifically, the indirect effect is more pronounced when the quality of change communication is high rather than when it is low.

**Figure 1:**
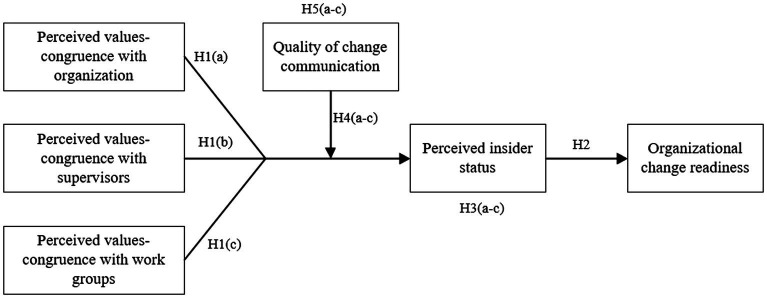
The proposed research model.

To sum up, we propose the following research framework (see Figure 1).

## 3. Research design

### 3.1. Sampling procedures

We carried out this study following the recommendations of the Ethics Committee of Hubei University of Economics with written informed consent from all subjects. To reduce the social desirability bias in the measurement for this study, we selected six companies that have recently undergone organizational change as the target research objects. Among these, three companies underwent departmental adjustments, two companies underwent structural adjustments for adopting new technology, and one company underwent a strategic transformation for shrinking business. The respondents to the survey were required to have worked in the current enterprise for no less than 5 years.

To ensure data quality and reduce the common method variance bias, we collected data at three time points with an interval of about 25 days each. The HR managers helped us deliver and recycle the questionnaires. Before conducting our surveys, we asked the HR managers from six different companies to offer us a coding list based on the employee rolls. Specifically, we gave an unique code to every company, every team supervisor, and every employee. For example, the first company was coded as C1, the supervisor in the first team of the roll was C1M01, and the first subordiate was coded as C1M01E01. In total, we got a 300 convenient sample size from 48 work teams. We conducted each survey during the monthly meetings.

At each stage, we distributed 300 questionnaires based on the coding list. If there is any respondent quit, the HR managers must also complete the coding information. In the first stage (Time 1), the volunteering respondents completed questionnaires including their basic demographic information (e.g., gender, age, education, tenure with the company) and perceived values-congruence. About 279 questionnaires were returned with a responding rate of 93%; in the second stage (Time 2), we also distributed 300 empployee questionnaires, the targeted respondents continued to finish PIS and the quality of change communication questionnaires. We got 258 completed questionnaires, due to some employees’ turnover or illness; in the last stage (Time 3), we distributed the questionnaires based on the coding list—the supervisor filled out the questionnaire regarding each participant’s organizational change readiness of the team. After excluding suspicious answers and missing data, we finally obtained 252 valid questionnaires that were successfully matched at different times.

Among these respondents, 54.4% are male, 21.4% are under 35 years old, and 52% of them have bachelor’s degrees. Analysis of variance indicated that there was no significant difference between the deleted sample and the valid sample in terms of their demographics (i.e., gender, age, and education level), and the item response bias was not obvious.

### 3.2. Measures

Due to the scales being derived from the English version, we adopted Brislin (1986) translation-and-back-translation procedure to ensure consistency of the content and meanings. All scales were five-point Likert scales ranging from “1 = strongly disagree” to “5 = strongly agree.”

#### 3.2.1. Perceived values-congruence

We measured PVC using the three-item scale of Cable and DeRue (2002). Specifically, we measured employees’ PVC with their organizations (PVC-O), with their supervisors (PVC-S), and with their workgroups (PVC-G), respectively. A sample item was “The things I value in my life are very similar to the things that my organization (supervisor, workgroup) values,” and Cronbach’s *α* values of PVC-O, PVC-S and PVC-G were 0.84, 0.79, and 0.88, respectively.

#### 3.2.2. Perceived insider status

We measured PIS using the five-item scale of Stamper and Masterson (2002). A sample item was “I feel very much a part of my work organization,” and Cronbach’s *α* value was 0.76.

#### 3.2.3. Quality of change communication

We measured QCC using the six-item scale of Edwards and Cable (2009). A sample item was “Always communicating openly with others about changes in the organization” and Cronbach’s *α* was 0.81.

#### 3.2.4. Organizational change readiness

We measured OCR using 28 items adopted from scales developed by Holt et al. (2007) and Bouckenooghe et al. (2009). The intermediate supervisor evaluated these scales about their subordinates. A sample item was “I think that the organization will benefit from this change,” and Cronbach’s *α* 0.83.

#### 3.2.5. Control variables

Following the recommendations of Bernerth and Aguinis (2016), we included the employee’s gender, age, education, and tenure with the organization as control variables. Prior studies have demonstrated that these variables are positively related to employees’ OCR (Metwally et al., 2019; Arneguy et al., 2022).

## 4. Data analysis

### 4.1. Preliminary analysis

Before testing our proposed hypotheses, we employed exploratory factor analysis to examine the common method bias (CMB) issue. Specifically, we conducted the Harman single-factor test by performing an unrotated factor solution for all variables. The first unrotated factor captured only 35.88% of the variance in data, which is less than the threshold value (50%), indicating that the CMB issue of the present study is not so severe in statistics.

Then, we conducted the confirmatory factor analysis on the data to test the discriminant validity of the six latent variable measures. The results are displayed in Table 1. Compared to other competing models, the fit indicators of the six-factor model excel all the other alternative models as the value of Chi-square changes is significant at the *p* < 0.001 level (*χ^2^/df* = 2.52, CFI = 0.93, NFI = 0.90, RMSEA = 0.09), indicating that the measurement has acceptable discriminant validity.

**Table 1:** Confirmatory factor analysis.

Models	^χ²^/df	RMSEA	CFI	NFI
Six-factor model: PVC-O; PVC-S; PVC-G; PIS; QCC; OCR	2.52	0.09	0.93	0.90
Five-factor model: PVC-O + PVC-S; PVC-G; PIS; QCC; OCR	3.37	0.11	0.87	0.84
Five-factor model: PVC-O; PVC-S + PVC-G; PIS; QCC; OCR	3.23	0.10	0.89	0.84
Four-factor model: PVC-O + PVC-S + PVC-G;PIS; QCC; OCR	4.37	0.13	0.83	0.81
Four-factor model: PVC-O + PVC-S; PVC-G + PIS;QCC;OCR	5.18	0.15	0.81	0.80
Three-factor model: PVCO + PVCS + PVC-G + QCC;PIS;OCR	5.90	0.18	0.77	0.76
Three-factor model: PVC-O + PVC-S + PVC-G; PIS; QCC + OCR	5.55	0.16	0.80	0.77
Two-factor model: PVC-O + PVC-S + PVC-G + PIS;QCC + OCR	6.28	0.19	0.78	0.77
Single-factor model: PVC-O + PVC-S + PVC-G + PIS + QCC + OCR	12.82	0.29	0.74	0.72

PVC-O, perceived values-congruence with organizations; PVC-S, perceived values-congruence with supervisors; PVC-G, perceived values-congruence with work groups; PIS, perceived insider status; QCC, quality of change communication; OCR, organizational change readiness

Table 2 displays the mean, standard deviation, and correlation coefficient each variable. As expected, employees’ perceived values-congruence with their organization (*r* = 0.23, *p* < 0.01), their supervisors (*r* = 0.33, *p* < 0.01), their work groups (*r* = 0.36, *p* < 0.01) are all positively related to their PIS, which is also positively related to their OCR (*r* = 0.24, *p* < 0.01). The analysis results provide preliminary support for hypotheses H1(a-c) and Hypothesis 2.

**Table 2:** Means, standard deviation, and correlations of variables.

Variables	1	2	3	4	5	6	7	8	9	10
1. Gender	1									
2. Age	−0.05	1								
3. Tenure with the organization	−0.02	0.09	1							
4. Education	0.01	0.12	−0.01	1						
5. PVC-O	0.03	0.08	0.14*	0.04	1					
6. PVC-S	0.07	0.01	0.07	0.02	0.52**	1				
7. PVC-G	−0.02	0.03	0.08	0.01	0.44**	0.49**	1			
8. PIS	−0.05	0.07	0.13*	−0.02	0.23**	0.33**	0.36**	1		
9. QCC	0.04	−0.05	0.06	0.01	0.06	0.02	0.12	0.17*	1	
10. OCR	0.01	0.07	0.11	0.04	0.38**	0.31**	0.40**	0.24**	0.14*	1
Means	0.59	3.08	2.37	1.39	3.71	3.44	3.77	3.81	3.14	3.49
SD	0.48	1.43	1.2	0.57	0.53	0.62	0.61	0.54	0.58	0.52

(i) *N* = 252, *p < 0.05; **p < 0.01; (ii) PVC-O, perceived values-congruence with organizations; PVC-S, perceived values-congruence with supervisors; PVC-G, perceived values-congruence with work groups; PIS, perceived insider status; QCC, quality of change communication; OCR, organizational change readiness

### 4.2. Hypotheses testing

Hypothesis 1(a–c) predicted employees’ perceived values-congruence with their organization (H1a), their supervisors (H1b), and their work groups (H1c) has a positive effect on PIS. As is displayed in Model1 of Table 3, perceived values-congruence (i.e., organizations, supervisors, and work groups) have a significant positive effect on employees’ PIS (β = 0.24, 0.31, and 0.33, respectively, all significant at the 0.01 level). Hence, Hypotheses 1a, 1b, and 1c are all well-supported.

**Table 3:** Multiple regression of multi-value congruence perception, perceived insider status, and organizational change readiness.

Variables	PIS				OCR		
	Model 1	Model 2	Model 3	Model 4	Model 5	Model 6	Model 7
Gender	0.02	0.01	0.01	0.02	0.00	0.02	0.00
Age	0.07	0.06	0.05	0.06	0.06	0.07	0.06
Tenure with organization	−0.04	0.11*	−0.06	0.02	0.05	0.04	−0.03
Education	0.04	0.03	0.04	0.05	−0.06	0.04	0.03
PVC-O	0.24**	0.10*			0.27**		0.23**
PVC-S	0.31**		0.21**		0.24**		0.22**
PVC-G	0.33**			0.27**	0.31**		0.26**
PIS						0.25**	0.15**
QCC		0.05	0.12*	0.14*			0.06
PVC-O × QCC		0.16**					0.09
PVC-S × QCC			0.09+				0.04
PVC-G × QCC				0.20**			0.15*
*R* ^2^	0.26	0.29	0.30	0.34	0.30	0.24	0.37

(i) *N* = 252, *p < 0.05; **p < 0.01; +*p* > 0.1; (ii) PVC-O, perceived values-congruence with organizations; PVC-S, perceived values-congruence with supervisors; PVC-G, perceived values-congruence with work groups; PIS, perceived insider status; QCC, quality of change communication; OCR, organizational change readiness.

Hypothesis 2 proposed that employees’ PIS positively affects OCR. As depicted in Model 6 of Table 3, PIS has a positive effect on employees’ OCR (β = 0.25, *p* < 0.01). Thereby, Hypothesis 2 is supported as well.

Hypothesis 3(a–c) predicted that PIS mediates the relationships between perceived values-congruence (i.e., congruence with their organizations, supervisors, and workgroups) and employees’ OCR. Hence, we conducted a bias-corrected bootstrapping test (*n* = 5,000). The results indicate that the indirect effect of PVC-O on OCR through PIS is 0.09 (95%CI = [0.02, 0.19]), excluding 0, and the mediating effect is significant; the indirect effect of PVC-S on OCR through PIS is 0.13 (95%CI = [0.05, 0.22]), excluding 0, and the mediating effect is significant; the indirect effect of PVC-G on OCR *via* PIS is 0.18 (95%CI = [0.08, 0.33]), which also does not contain 0, indicating that the mediating effect is significant. Therefore, hypotheses 3a, 3b, and 3c are all verified.

Hypothesis 4(a–c) proposed that employees’ perceived values-congruence with their organization (H4a), their supervisors (H4b), and their work groups (H4c) have a more substantial positive effect on PIS when the quality of change communication is high rather than when it is low. As demonstrated in Model 2 and Model 4 in Table 3, the effect of the interaction between PVC-O and QCC on PIS is significant (β = 0.16, *p* < 0.01). In the same vein, the interplay between PVC-G and QCC on PIS is also significant (β = 0.20, *p* < 0.01), signifying that the higher the quality of change communication, the stronger the positive effects of PVC-O and PVC-G on PIS. However, the interaction coefficient of PVC-S and QCC on PIS is not significant (β = 0.09, *p* < 0.10), and hence cannot thoroughly verify the moderating effect of change communication quality in the relationship between PVC-S and PIS. Therefore, Hypotheses 4a and 4c are entirely substantiated, whereas Hypothesis 4b is only partially substantiated.

Hypothesis 5(a–c) predicted that the quality of change communication could moderate the indirect effects of PIS between employees’ perceived values-congruence with their organization (5a), their supervisors (5b), and their work groups (5c) on employees’ OCR. We also conducted moderated mediation analysis, and the results suggested that the quality of change communication significantly moderated the impact of PVC-O on OCR through PIS (β = 0.08, 95%CI = [0.04, 0.21]). Specifically, when the quality of change communication was low (−1 *SD*), the indirect effect of PIS was 0.03 (95%CI = [−0.00, 0.16]), including 0, and hence the mediating effect was not significant; when the quality of change communication was high (+1 *SD*), the indirect effect was 0.13 (95%CI = [0.04, 0.27]), and hence the mediating effect was significant. The difference between the high and low groups was 0.10 (95%CI = [0.02, 0.31]), excluding 0, and the difference was significant. Thus, Hypothesis 5a is verified.

Likewise, the quality of change communication significantly moderated the effect of PVC-S on OCR through PIS (β = 0.17, 95%CI = [0.07, 0.38], excluding 0). Specifically, when the quality of change communication was low (−1 *SD*), the indirect effect of PIS was −0.00 (95%CI = [−0.00, 0.09]), containing 0, whereas when the quality of change communication was high (+1 *SD*), the indirect effect of PIS was 0.19 (95% CI = [0.06, 0.40]). The difference between the high and low groups was 0.20 (95%CI = [0.02, 0.36]), excluding 0. Thus, Hypothesis 5c was verified. However, the indirect moderating effect of the quality of change communication on the PVC-S on OCR through PIS is not significant (β = 0.03, 95%CI = [−0.02, 0.11], including 0. Therefore, Hypothesis 5b is not verified.

## 5. Discussion

This study aims to advance the growing body of literature regarding the effects and mechanisms of perceived values-congruence with their organizations, supervisors, and workgroups (PVC-O, PVC-S, and PVC-G) on OCR (e.g., Rahn et al., 2020). Data from 252 employees in six companies supports most of the paths of our hypothesized model. Overall, we find a positive relationship between perceived values-congruence (PVC-O, PVC-S, and PVC-G) and PIS. Further, we demonstrate that employees’ PIS mediates the impact of employees’ perceived values-congruence on their readiness for organizational change. Besides, we further found that the mediation effect of PIS is dependent on the quality of change communication. Specifically, with high-quality change communication, employees’ PVC-O and PVC-G significantly positively contribute to employees’ OCR *via* PIS, whereas the moderating effect of change communication quality on PVC-S and PIS is not supported by statistical analysis. One possible explanation might be that the supervisors are always the agent in determining the quality of change communication, no matter whether or how much the supervisors communicate change-related information to them, the employees will follow their supervisors who share similar values on organizational change. Our findings extend and enrich the literature on the relationship between values-congruence and organizational change readiness and have important implications for management practice. Next, we elaborate on the theoretical contributions and practical implications of our findings.

### 5.1. Theoretical contributions

The present study makes several theoretical contributions to the literature by unraveling the underlying mechanism of PIS in driving the relationship between perceived values-congruence and employees’ OCR. Firstly, to our knowledge, the present study is one of the first few empirical research to show the impact of perceived values-congruence on OCR. OCR is formed through a complex process influenced by many factors. Although existing studies have made valuable attempts at examining antecedents of OCR from multiple perspectives and dimensions (Burnes and Jackson, 2011). For example, studies have found that leadership support (Soumyaja et al., 2011), trust and change participation (Shah and Ghulam Sarwar Shah, 2010), and organizational justice (Arneguy et al., 2020; Kebede and Wang, 2022) positively impact organizational change readiness. Yet, scant attention has been paid to the impact of values-congruence on OCR.

Secondly, we extend the research on why value congruence can foster employees’ OCR. Previous research has indicated that value congruence has a positive effect on employees’ OCR (Branson, 2008; Alas, 2009; Rahn et al., 2020; Tang et al., 2021), yet not many scholars paid attention to the underlying mechanisms. The present study offers an additional plausible explanation (i.e., PIS). In contrast, Rahn et al. (2020) demonstrated trust as an underlying mechanism in explaining the effect of employees’ perceived values-congruence on organizational change readiness. Although both trust and PIS can be cognitive mechanisms, the former aligns with the social exchange perspective, whereas the latter lies in the self-categorization perspective.

Finally, the moderation findings regarding the quality of change communication respond to the scholarly calls for exploring the boundary conditions on the relationships of value congruence with its outcomes (Rogiest et al., 2015; Rahn et al., 2020). Specifically, Prior research has highlighted employees’ access to comprehensive, detailed, specific, and timely change information can help reduce change anxiety and feelings of stress, enhance change perceptions, and lead to a better response to change challenges (Stevens, 2013; Neill et al., 2020). This study demonstrates that the quality of change communication plays a crucial role in the effect that employees’ perceived values-congruence has on organizational change readiness, echoing and extending findings from previous studies. This study found that high-quality change communication can strengthen psychological suggestion through “information influence,” usher in a stronger PIS, and thus improve employees’ OCR; on the contrary, low-quality change communication weakens the positive effect of employees’ perceived values-congruence on PIS and OCR.

### 5.2. Practical implications

Our research findings provide several managerial insights into cultivating employees’ OCR. First of all, our study demonstrates that values-congruence plays a crucial role in promoting the organizational change readiness of the employees. Accordingly, managers need to pay increased attention to value management while implementing organizational change and regard value congruence as an essential condition and pre-measure for planned change. Secondly, our results indicate that PIS, a critical positive psychological state, precedes the employees’ OCR. Hence, keeping track of followers’ PIS is an effective way for supervisors to determine whether values congruence can ultimately enhance followers’ OCR.

Finally, our findings suggest that the quality of change communication is a critical that can influence the effectiveness of values-congruence on OCR. Hence, the management should prioritize change communication as a vital strategy and ensure its quality. Specifically, in the entire organizational change process, the management should make the change information open and transparent, contain information conducive to the formation of organizational attachment and psychological belonging, and integrate the value image of the organization’s future expectations into the change message.

### 5.3. Limitations and future research

Although this study makes a few theoretical contributions, it has certain drawbacks.

Firstly, we only collected the sample from six companies, and the respondents answered the questionnaires retrospectively (Bono and McNamara, 2011). The limitations of the sample could affect the external validity of the present study, and the loss of information caused by the passage of time, such as vague memory and lost episodes, could also lead to biased findings. Similarly, although this study adopted time-specific data collection to reduce homogeneous method bias to a certain extent, it could not eliminate possible interactions between variables because not all variables were measured simultaneously. To this end, future research can draw on longitudinal research designs to simultaneously track and measure the related organizational change issues at multiple time points or adopt experimental methods for causal inference.

Secondly, this study assumes employees’ perceived values-congruence as the antecedent of organizational change readiness and incorporates change management strategies—change communication quality and acquaintances effects—into the analysis framework. This study verifies the positive role of perceived values-congruence in producing organizational change readiness. Based on the premise of differentiating between the nature and content of organizational change, the role of values-congruence on employees’ responses to change may vary subtly (Branson, 2008). Employees are more likely to avoid or even resist change if they believe that their previous state of alignment with organizational values would be terminated or even destroyed as a result of organizational change (Zhang et al., 2020); however, if employees expect a greater convergence of organizational values and self-values after the change, they will demonstrate a higher commitment to and enthusiasm for change (Neves and Caetano, 2009). Moreover, during different stages of organizational change, differences are apparent in employees’ organizational trust, psychological belonging, and identity (Choi, 2011; Bouckenooghe et al., 2021). Therefore, future research could distinguish between different organizational change stages and examine the double-edged effect of perceived values-congruence on the various facets of organizational change.

## 6. Conclusion

Integrating the self-categorization theory and social information processing theory, we provide clear evidence that employees’ perceived values-congruence is a precursor for organizational change readiness. Specifically, the employees’ values-congruence (i.e., employee-organization, employee-supervisor, and employee-workgroup) is a critical determinant for successfully implementing organizational change, hence needing sufficient attention. Then, the cultivation of PIS is an essential mechanism for cultivating employees’ organizational change readiness. Further, we demonstrate that the quality of change communication is a critical contingent factor that can influence the effects of perceived values-congruence on PIS. Therefore, these factors should not be neglected. Practically, we provide new insight into leveraging the positive effects of perceived values-congruence on promoting employees’ OCR.

## Data availability statement

The raw data supporting the conclusions of this article will be made available by the authors, without undue reservation.

## Author contributions

JD was the principal investigator of the grants and finalized the manuscript. ZC analyzed the data, revised the draft, and improved the manuscript. SQ and RD helped with the research design and data collection. All authors contributed to the article and approved the submitted version.

## Funding

This research was supported by the National Natural Science Foundation of China (grant nos. 71472060 and 72202096) and Humanity and Social Science Foundation of the Ministry of Education of China (grant no. 20YJA630010).

## Conflict of interest

The authors declare that the research was conducted in the absence of any commercial or financial relationships that could be construed as a potential conflict of interest.

## Publisher’s note

All claims expressed in this article are solely those of the authors and do not necessarily represent those of their affiliated organizations, or those of the publisher, the editors and the reviewers. Any product that may be evaluated in this article, or claim that may be made by its manufacturer, is not guaranteed or endorsed by the publisher.

## References

[ref1] Alas R. (2009). The impact of work-related values on the readiness to change in Estonian organizations. J. Bus. Ethics 86, 113–124. doi: 10.1007/s10551-008-9838-5

[ref2] Alavi S. B. Gill C. (2017). Leading change authentically: how authentic leaders influence follower responses to complex change. J. Leadersh. Organ. Stud. 24, 157–171. doi: 10.1177/1548051816664681

[ref3] Aldabbas H. (2022). Antecedents and consequences of perceived insider status and suggestions for future research. J. Pos. School Psychol. 6, 2813–2832.

[ref4] Arneguy E. Ohana M. Stinglhamber F. (2020). Overall justice, perceived organizational support and readiness for change: the moderating role of perceived organizational competence. J. Organ. Chang. Manag. 33, 765–777. doi: 10.1108/JOCM-12-2019-0373

[ref5] Arneguy E. Ohana M. Stinglhamber F. (2022). Readiness for change: which source of justice and support really matters? Empl. Relat. 44, 210–228. doi: 10.1108/ER-05-2020-0225

[ref6] Augustsson H. Richter A. Hasson H. Schwarz U. V. T. (2017). The need for dual openness to change: a longitudinal study evaluating the impact of employees’ openness to organizational change content and process on intervention outcomes. J. Appl. Behav. Sci. 53, 349–368. doi: 10.1177/0021886317691930

[ref7] Bakari H. Hunjra A. I. Niazi G. S. K. (2017). How does authentic leadership influence planned organizational change? The role of employees’ perceptions: integration of theory of planned behavior and Lewin’s three step model. J. Chang. Manag. 17, 155–187. doi: 10.1080/14697017.2017.1299370

[ref8] Bennett N. Lemoine G. J. (2014). What a difference a word makes: understanding threats to performance in a VUCA world. Bus. Horiz. 57, 311–317. doi: 10.1016/j.bushor.2014.01.001

[ref9] Bernerth J. (2004). Expanding our understanding of the change message. Hum. Resour. Dev. Rev. 3, 36–52. doi: 10.1177/1534484303261230

[ref10] Bernerth J. B. Aguinis H. (2016). A critical review and best-practice recommendations for control variable usage. Pers. Psychol. 69, 229–283. doi: 10.1111/peps.12103

[ref11] Bono J. E. McNamara G. (2011). Publishing in AMJ—part 2: research design. Acad. Manag. J. 54, 657–660. doi: 10.5465/amj.2011.64869103

[ref12] Borges R. Quintas C. A. (2020). Understanding the individual’s reactions to the organizational change: a multidimensional approach. J. Organ. Chang. Manag. 33, 667–681. doi: 10.1108/JOCM-09-2019-0279

[ref13] Bouckenooghe D. De Clercq D. Deprez J. (2014). Interpersonal justice, relational conflict, and commitment to change: the moderating role of social interaction. Appl. Psychol. 63, 509–540. doi: 10.1111/apps.12006

[ref14] Bouckenooghe D. Devos G. Van den Broeck H. (2009). Organizational change questionnaire-climate of change, processes, and readiness: development of a new instrument. J. Psychol. 143, 559–599. doi: 10.1080/00223980903218216, PMID: 19957876

[ref15] Bouckenooghe D. Schwarz G. M. Kanar A. Sanders K. (2021). Revisiting research on attitudes toward organizational change: bibliometric analysis and content facet analysis. J. Bus. Res. 135, 137–148. doi: 10.1016/j.jbusres.2021.06.028

[ref16] Branson C. M. (2008). Achieving organizational change through values alignment. J. Educ. Adm. 46, 376–395. doi: 10.1108/09578230810869293

[ref17] Brislin R. W. (1986). “The wording and translation of research instruments” in Field methods in cross-cultural research. eds. Lonner W. J. Berry J. W. (Beverly Hills, CA: Sage), 137–164.

[ref18] Burnes B. Jackson P. (2011). Success and failure in organizational change: an exploration of the role of values. J. Organ. Chang. Manag. 11, 133–162. doi: 10.1080/14697017.2010.524655

[ref19] Cable D. M. DeRue D. S. (2002). The convergent and discriminant validity of subjective fit perceptions. J. Appl. Psychol. 87, 875–884. doi: 10.1037/0021-9010.87.5.875, PMID: 12395812

[ref20] Choi M. (2011). Employees’ attitudes toward organizational change: a literature review. Hum. Resour. Manag. 50, 479–500. doi: 10.1002/hrm.20434

[ref21] Edwards J. R. Cable D. M. (2009). The value of value congruence. J. Appl. Psychol. 94, 654–677. doi: 10.1037/a0014891 19450005

[ref22] Edwards J. R. Lambert L. S. (2007). Methods for integrating moderation and mediation: a general analytical framework using moderated path analysis. Psychol. Methods 12, 1–22. doi: 10.1037/1082-989X.12.1.1, PMID: 17402809

[ref23] Gfrerer A. Hutter K. Fuller J. Strohle T. (2021). Ready or not: managers’ and employees’ different perceptions of digital readiness. Calif. Manag. Rev. 63, 23–48. doi: 10.1177/0008125620977487

[ref24] Güntner A. V. Endrejat P. C. Kauffeld S. (2021). The emergence of employees’ change readiness for energy-conservation behavior during guided group discussions. Front. Psychol. 12:587529. doi: 10.3389/fpsyg.2021.587529, PMID: 34790140 PMC8591390

[ref25] Hameed I. Khan A. K. Sabharwal M. Arain G. A. Hameed I. (2019). Managing successful change efforts in the public sector: an Employee’s readiness for change perspective. Rev. Pub. Pers. Admin. 39, 398–421. doi: 10.1177/0734371X17729869

[ref26] Hoffman B. J. Bynum B. H. Piccolo R. F. Sutton A. W. (2011). Person-organization value congruence: how transformational leaders influence work group effectiveness. Acad. Manag. J. 54, 779–796. doi: 10.5465/amj.2011.64870139

[ref27] Holt D. T. Armenakis A. A. Feild H. S. Harris S. G. (2007). Readiness for organizational change: the systematic development of a scale. J. Appl. Behav. Sci. 43, 232–255. doi: 10.1177/0021886306295295

[ref28] Hui C. Lee C. Wang H. (2015). Organizational inducements and employee citizenship behavior: the mediating role of perceived insider status and the moderating role of collectivism. Hum. Resour. Manag. 54, 439–456. doi: 10.1002/hrm.21620

[ref29] Judge T. A. Thoresen C. J. Pucik V. Welbourne T. M. (1999). Managerial coping with organizational change: a dispositional perspective. J. Appl. Psychol. 84, 107–122. doi: 10.1037/0021-9010.84.1.107

[ref30] Kebede S. Wang A. (2022). Organizational justice and employee readiness for change: the mediating role of perceived organizational support. Front. Psychol. 13:806109. doi: 10.3389/fpsyg.2022.806109, PMID: 35369209 PMC8965650

[ref31] Kristof-Brown A. L. Zimmerman R. D. Johnson E. C. (2005). Consequences OF INDIVIDUALS’FIT at work: a meta-analysis OF person–job, person–organization, person–group, and person–supervisor fit. Pers. Psychol. 58, 281–342. doi: 10.1111/j.1744-6570.2005.00672.x

[ref32] Kwahk K.-Y. Kim H.-W. (2008). Managing readiness in enterprise systems-driven organizational change. Behav. Inform. Technol. 27, 79–87. doi: 10.1080/01449290701398475

[ref33] Lamm E. Gordon J. R. Purser R. E. (2010). The role of value congruence in organizational change. Organ. Dev. J. 28, 49–64.

[ref34] Metwally D. Ruiz-Palomino P. Metwally M. Gartzia L. (2019). How ethical leadership shapes employees’ readiness to change: the mediating role of an organizational culture of effectiveness. Front. Psychol. 10:2493. doi: 10.3389/fpsyg.2019.02493, PMID: 31798489 PMC6874171

[ref35] Naumtseva E. A. Stroh W. A. (2020). Psychological readiness for organizational change and its socio-psychological predictors. Soc. Psychol. Soc. 11, 151–164. doi: 10.17759/sps.2020110411

[ref01] Neves P. Caetano A. (2009). Commitment to change: Contributions to trust in the supervisor and work outcomes. Group and Organization Management 34, 623–644. doi: 10.1177/1059601109350980

[ref36] Neill M. S. Men L. R. Yue C. A. (2020). How communication climate and organizational identification impact change. Corp. Commun. Int. J. 25, 281–298. doi: 10.1108/CCIJ-06-2019-0063

[ref37] Posner B. Z. (2010). Another look at the impact of personal and organizational values congruency. J. Bus. Ethics 97, 535–541. doi: 10.1007/s10551-010-0530-1

[ref38] Rafferty A. E. Jimmieson N. L. Armenakis A. A. (2013). Change readiness: a multilevel review. J. Manag. 39, 110–135. doi: 10.1177/0149206312457417

[ref39] Rafferty A. E. Minbashian A. (2019). Cognitive beliefs and positive emotions about change: relationships with employee change readiness and change-supportive behaviors. Hum. Relat. 72, 1623–1650. doi: 10.1177/0018726718809154

[ref40] Rahn O. G. Soutar G. N. Lee J. A. (2020). Perceived values-congruence and employees’ change beliefs. J. Manag. Organ. 1-19, 1–19. doi: 10.1017/jmo.2020.4

[ref41] Roemer A. Sutton A. Medvedev O. N. (2021). The role of dispositional mindfulness in employee readiness for change during the COVID-19 pandemic. J. Organ. Chang. Manag. 34, 917–928. doi: 10.1108/JOCM-10-2020-0323

[ref42] Rogiest S. Segers J. van Witteloostuijn A. (2015). Climate, communication and participation impacting commitment to change. J. Organ. Chang. Manag. 28, 1094–1106. doi: 10.1108/JOCM-06-2015-0101

[ref43] Salancik G. R. Pfeffer J. (1978). A social information processing approach to job attitudes and task design. Adm. Sci. Q. 23, 224–253. doi: 10.2307/2392563, PMID: 10307892

[ref44] Santhidran S. Chandran V. Borromeo J. (2013). Enabling organizational change–leadership, commitment to change and the mediating role of change readiness. J. Bus. Econ. Manag. 14, 348–363. doi: 10.3846/16111699.2011.642083

[ref45] Seggewiss B. J. Straatmann T. Hattrup K. Mueller K. (2019). Testing interactive effects of commitment and perceived change advocacy on change readiness: investigating the social dynamics of organizational change. J. Chang. Manag. 19, 122–144. doi: 10.1080/14697017.2018.1477816

[ref46] Shah N. Irani Z. Sharif A. M. (2017). Big data in an HR context: exploring organizational change readiness, employee attitudes and behaviors. J. Bus. Res. 70, 366–378. doi: 10.1016/j.jbusres.2016.08.010

[ref47] Shah N. Ghulam Sarwar Shah S. (2010). Relationships between employee readiness for organizational change, supervisor and peer relations and demography. J. Enterp. Inf. Manag. 23, 640–652. doi: 10.1108/17410391011083074

[ref48] Smollan R. K. Sayers J. G. (2009). Organizational culture, change and emotions: a qualitative study. J. Chang. Manag. 9, 435–457. doi: 10.1080/14697010903360632

[ref49] Soumyaja D. Kamalanabhan T. Bhattacharyya S. (2011). Employee readiness to change and individual intelligence: the facilitating role of process and contextual factors. Int. J. Bus. Insights Trans. 4, 85–92.

[ref50] Stamper C. L. Masterson S. S. (2002). Insider or outsider? How employee perceptions of insider status affect their work behavior. J. Organ. Behav. 23, 875–894. doi: 10.1002/job.175

[ref51] Stevens G. W. (2013). Toward a process-based approach of conceptualizing change readiness. J. Appl. Behav. Sci. 49, 333–360. doi: 10.1177/0021886313475479

[ref52] Stouten J. Rousseau D. M. De Cremer D. (2018). Successful organizational change: integrating the management practice and scholarly literatures. Acad. Manag. Ann. 12, 752–788. doi: 10.5465/annals.2016.0095

[ref53] Tang J. Mo L. Liu W.-B. (2021). The attributes of organizational change: how person-organization value congruence influences employees’ coping. J. Organ. Chang. Manag. 34, 121–136. doi: 10.1108/JOCM-04-2017-0122

[ref54] Tanner G. Otto K. (2016). Superior–subordinate communication during organizational change: under which conditions does high-quality communication become important? Int. J. Hum. Resour. Manag. 27, 2183–2201. doi: 10.1080/09585192.2015.1090470

[ref55] Thakur R. R. Srivastava S. (2018). From resistance to readiness: the role of mediating variables. J. Organ. Chang. Manag. 31, 230–247. doi: 10.1108/JOCM-06-2017-0237

[ref56] Turner J. C. Hogg M. A. Oakes P. J. Reicher S. D. Wetherell M. S. (1987). Rediscovering the social group: A self-categorization theory. Oxford, New York, NY: Basil Blackwell.

[ref57] Wang J. Kim T. Y. (2013). Proactive socialization behavior in China: the mediating role of perceived insider status and the moderating role of supervisors’ traditionality. J. Organ. Behav. 34, 389–406. doi: 10.1002/job.1811

[ref58] Xiao J. Mao J.-Y. Huang S. Qing T. (2020). Employee-organization fit and voluntary green behavior: a cross-level model examining the role of perceived insider status and green organizational climate. Int. J. Environ. Res. Public Health 17:2193. doi: 10.3390/ijerph17072193, PMID: 32218284 PMC7177816

[ref59] Zhang Y. Sun J. Yang Z. Wang Y. (2020). Critical success factors of green innovation: technology, organization and environment readiness. J. Clean. Prod. 264:121701. doi: 10.1016/j.jclepro.2020.121701

